# Eng2, a new player involved in feedback loop regulation of Cdc42 activity in fission yeast

**DOI:** 10.1038/s41598-021-97311-6

**Published:** 2021-09-09

**Authors:** Patricia García, Pedro M. Coll, Francisco del Rey, M. Isabel Geli, Pilar Pérez, Carlos R. Vázquez de Aldana, Javier Encinar del Dedo

**Affiliations:** 1 Instituto de Biología Funcional y Genómica, CSIC/Universidad de Salamanca, c/ Zacarías González 2, 37007 Salamanca, Spain; 2 Institute for Molecular Biology of Barcelona (CSIC), Baldiri Reixac 15, 08028 Barcelona, Spain

**Keywords:** Cell biology, Cell polarity

## Abstract

Cell polarity and morphogenesis are regulated by the small GTPase Cdc42. Even though major advances have been done in the field during the last years, the molecular details leading to its activation in particular cellular contexts are not completely understood. In fission yeast, the β(1,3)-glucanase Eng2 is a “moonlighting protein” with a dual function, acting as a hydrolase during spore dehiscence, and as component of the endocytic machinery in vegetative cells. Here, we report that Eng2 plays a role in Cdc42 activation during polarized growth through its interaction with the scaffold protein Scd2, which brings Cdc42 together with its guanine nucleotide exchange factor (GEF) Scd1. *eng2*Δ mutant cells have defects in activation of the bipolar growth (NETO), remaining monopolar during all the cell cycle. In the absence of Eng2 the accumulation of Scd1 and Scd2 at the poles is reduced, the levels of Cdc42 activation decrease, and the Cdc42 oscillatory behavior, associated with bipolar growth in wild type cells, is altered. Furthermore, overexpression of Eng2 partially rescues the growth and polarity defects of a *cdc42-L160S* mutant. Altogether, our work unveils a new factor regulating the activity of Cdc42, which could potentially link the polarity and endocytic machineries.

## Introduction

Polarized growth is essential for morphogenesis and completion of a correct developmental program in eukaryotic organisms reviewed in Refs.^1–5^. Defects in polarized growth lead to different diseases such as neuronal disorders or cancer ^6–10^.

In *Schizosaccharomyces pombe*, polarized growth is limited to the cell tips during vegetative growth and to the mating projections during conjugation reviewed in Refs.^4,11,12^. After cell separation, a new-born cell initially grows at a single pole (the old pole opposite to the cytokinesis site), and later, in G2 phase, it undergoes a cell cycle-regulated switch termed New End Take-Off (NETO), which creates two growth poles until mitosis^13^. Genetic analyses identified numerous factors that affect the establishment of cell polarity and NETO ^14–16^. Thus, the Tea1–Tea4 complex acts as a polarity marker, since mutants lacking these proteins produce cells with a “T-Shape” morphology. Tea4 interacts with the formin For3, which is required for interphase actin cable formation and whose regulation is mediated by proteins like Cdc42, Pob1 or Bud6 ^17–19^. Cdc42 is a key element in the regulation of polarized growth in eukaryotic cells reviewed in Refs.^2,20,21^. This small GTPase is essential for viability in *S. pombe*. Distinct mutant alleles cause different defects in growth, secretion, endosomal traffic, vacuole formation or actin cable organization, probably reflecting impaired interaction with particular effectors^22–24^. Cdc42 is regulated by two GEFs, Gef1 and Scd1 ^25–27^, which share an essential function but have different functions in polarized growth ^26^. Scd1 is required for establishing polarity and needs the scaffold protein Scd2 to interact with Cdc42 ^27^. Scd1 also restricts Gef1 localization to maintain the morphology during vegetative growth ^27–29^. Gef1 is dispensable for cell polarization per se but promotes Scd1 recruitment to the new end to enable the transition from monopolar to bipolar growth ^27,30^. Cdc42 activation displays a remarkable oscillatory behavior during bipolar growth in *S. pombe* ^30^, which derive from amplification of a small initial stochastic event through positive feedback mechanisms ^31–33^.

Polarized growth is strongly linked to secretion and endocytosis ^12,22,34–36^. Cdc42 and phosphatidylinositol 4,5-bisphosphate (PI(4,5)P_2_) define the position of the exocyst complex ^37^. Reciprocally, polarized secretion is required to maintain cell polarity ^38,39^. Mutants in different exocyst subunits have morphological defects that are exacerbated when actin cables are disrupted ^40,41^. Similarly, mutations in endocytic proteins like End4/Sla2, Myo1 or Btn1 install defects in cell polarity in *S. pombe* ^42–44^, *S. cerevisiae* ^45,46^ and mammalian cells ^47^.

Eng2 is a “moonlighting protein” with different functions along the cell cycle. During sporulation, it functions as a β(1,3)-glucanase required to hydrolyze the ascus wall allowing the release of ascospores ^48^. During vegetative growth, Eng2 forms a cytoplasmic complex with Lsb1/Csh3 that is necessary for coupling the endocytic coat to the actin module during endocytosis ^49^. In *eng2*Δ cells, endocytosis is blocked at high temperature and cells show growth defects and rounded morphology, indicative of a defect in polarity establishment or maintenance ^49^.

Here, we show that in addition to those functions, *eng2∆* cells have a defect in NETO establishment. We have found that Eng2 interacts with the Cdc42 scaffold protein Scd2, and that depletion of Eng2 causes a reduction in the tip accumulation of Scd2 and the Cdc42 GEF Scd1. Probably as a consequence of the Scd2-Scd1 localization defect, the cellular pool of active Cdc42 drops in the absence of Eng2, and its oscillatory behavior is altered, explaining the defects in polarized growth and morphogenesis of *eng2∆* cells.

Altogether, our work unveils a new element interacting with a component of the functional network of Cdc42 and regulates its GTPase activity directly or indirectly, which could potentially link the polarity and endocytic machineries.

## Results

### *eng2*Δ mutant cells have a defect in cell polarity establishment

**Figure 1:**
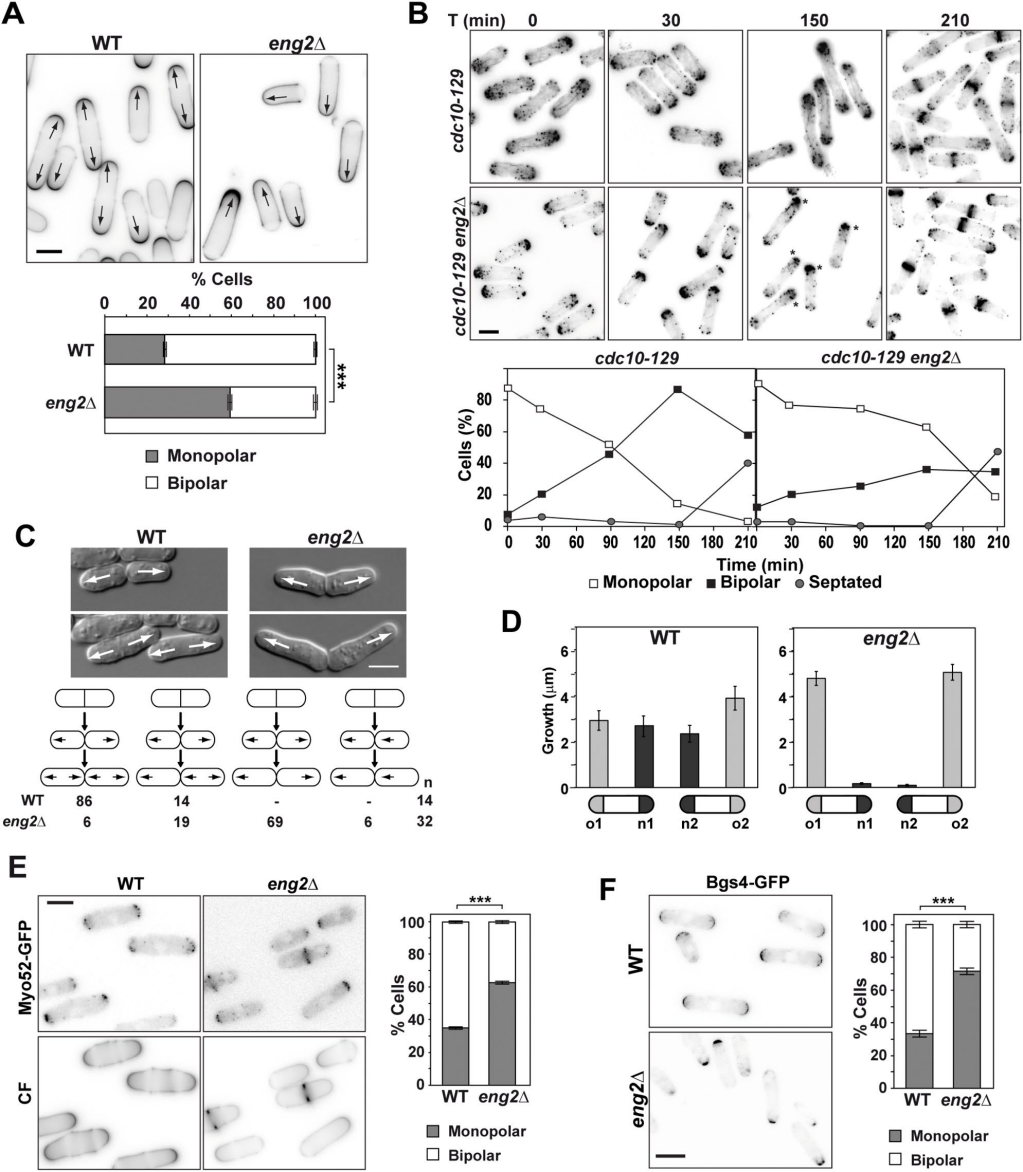
*eng2*Δ cells have NETO defects. **(A)** Calcofluor white staining of wild-type and *eng2*Δ cells grown in EMM medium. Arrows indicate the growing poles. Scale bar, 5 μm. The graph represents the average ± s.e.m. percentage of monopolar or bipolar cells in each strain of 3 independent experiments (n > 150; ***p-value < 0.0001). **(B)** *cdc10-129* and *cdc10-129 eng2*Δ cells were arrested in G1 as monopolar cells by incubation at 36 °C and then released at 25 °C. Images show actin staining with Alexa Fluor 488 phalloidin at the indicated times (min) after release. Asterisks indicate cells with monopolar growth. The graphs represent the percentage of monopolar, bipolar and septated cells at each point. Scale bar, 5 μm. **(C)** Identification and quantification of growth patterns of wild-type and *eng2*Δ cells by time-lapse microscopy. Arrows indicate the direction of growth. The percentage of cells with each growth pattern is indicated. Scale bar, 5 μm. **(D)** Total growth for each cell tip during the cell cycle for wild-type (n = 12) and in *eng2*Δ (n = 22) daughter cell pairs (average ± s.e.m). Cells were grown on EMM during 4–5 h at 28 °C. **(E)** Localization of Myo52-GFP in wild-type and *eng2*Δ cells. Images of Myo52-GFP and calcofluor staining (CF) in the same cells are shown (scale bar, 5 μm). The graph represents the average ± s.e.m. percentage of cells with monopolar or bipolar localization of Myo52-GFP in each strain for 3 independent experiments (n > 150; ***p-value < 0.0001). **(F)** Localization of Bgs4-GFP in wild-type and *eng2*Δ cells. Fluorescence images are shown (scale bar, 5 μm). The graph represents the average ± s.e.m. percentage of cells with monopolar or bipolar Bgs4-GFP in each strain for 3 independent experiments (n > 150; ***p-value < 0.0001).

Eng2 is a protein with dual function: it has β(1,3)-glucanase activity required for the hydrolysis of the ascus cell wall after spore formation has been completed ^48^ and it also functions during vegetative growth as a component of an endocytic module that couples the endocytic coat to the actin cytoskeleton ^49^. In addition to defects in endocytic uptake, we noticed that *eng2*Δ mutants have defects in establishing growth at the new end, as judged by calcofluor white staining of logarithmic-growing *eng2*Δ cells. Calcofluor marks *S. pombe* septum and growth regions. In asynchronous cultures, around 60–70% of wild type cells were bipolar whereas the percentage decreased to 30–40% of *eng2*Δ cells (Fig. 1A), indicating that *eng2*^+^ is required for growth activation at the new pole (NETO).

To confirm this observation, *cdc10-129* and *cdc10-129 eng2*Δ strains were used to synchronize the cells by arrest-release and then, the actin cytoskeleton was stained with phalloidin. When cultured at 36 °C, *cdc10-129* cells arrest in G1, and cell growth is polarized to one of the tips ^50^. In the *cdc10-129* strain, bipolar cells appeared 30 min after the release from the block, reaching a maximum of 80–90% of the culture at 150 min, while only 35% of *cdc10-129 eng2*Δ cells were able to switch to bipolar growth (Fig. 1B). The timing of septum formation was similar in both strains, suggesting that *eng2*^+^ deletion did not delay cell cycle progression. Together, these results indicated that Eng2 had a function in growth activation at the new end.

The growth pattern of *eng2*Δ cells was further analyzed by time-lapse microscopy. After cell division, both daughter cells activated growth from the old end and then switched to bipolar growth in the wild-type strain (Fig. 1C). In contrast, in the *eng2*Δ mutant strain the majority of daughter cells (69%) initiated cell growth at the old end and never activated growth at the new end, consistent with the calcofluor staining pattern. Interestingly, only a small percentage (6%) of cells had a growth pattern similar to that of *tea1*Δ or *tea4*Δ mutants, i.e. one cell grew at the old end and the other from the new end ^14,51^ suggesting that *eng2*Δ cells do not have a deffect in re-establishing growth. Total growth for each cell tip in wild-type and *eng2*Δ daughter cell pairs was determined from time-lapse analysis and confirmed the differences in the growth pattern between the two strains (Fig. 1D). Additionally, we quantified the growth rate of WT and *eng2Δ* cells and we found significant differences at both poles (Fig S1A), consistent with the above observations. When total growth was determined in both strains, it was slightly higher in wild type cells (5.92 ± 1.52 μm versus 5.04 ± 0.85 μm in *eng2*Δ cells), explaining the slightly smaller size of mutant cells. Together, these results suggest that Eng2 is required for growth activation at the new end and that it functions in a pathway different from that of the polarity markers Tea1 and Tea4.

To investigate the possible nature of the NETO defect, we analyzed the localization of proteins required for polarity establishment and for polarized growth in *eng2*Δ cells. No defects were observed in the localization of the Tea1 polarity marker ^52^, as compared to wild-type cells (Fig. S1B). This indicates that Eng2 functions downstream of this protein. However, other proteins required for polarized growth -such as the class V myosin Myo52^53^—were mainly localized to one of the poles (Fig. 1E). The percentage of cells with monopolar Myo52 distribution in the mutant perfectly matched the percentage of monopolar *eng2*Δ cells. Indeed, Myo52 always localized to the growing pole of cells. Thus, the absence of Myo52 in the new ends could be related with the previously described defect in the directionality of the actin cables ^49^. Consistently, the Bgs4 subunit of the glucan synthase^54^ also had a monopolar distribution in the *eng2*Δ mutant (Fig. 1F).

### Absence of Eng2 reduces the levels of active Cdc42

**Figure 2:**
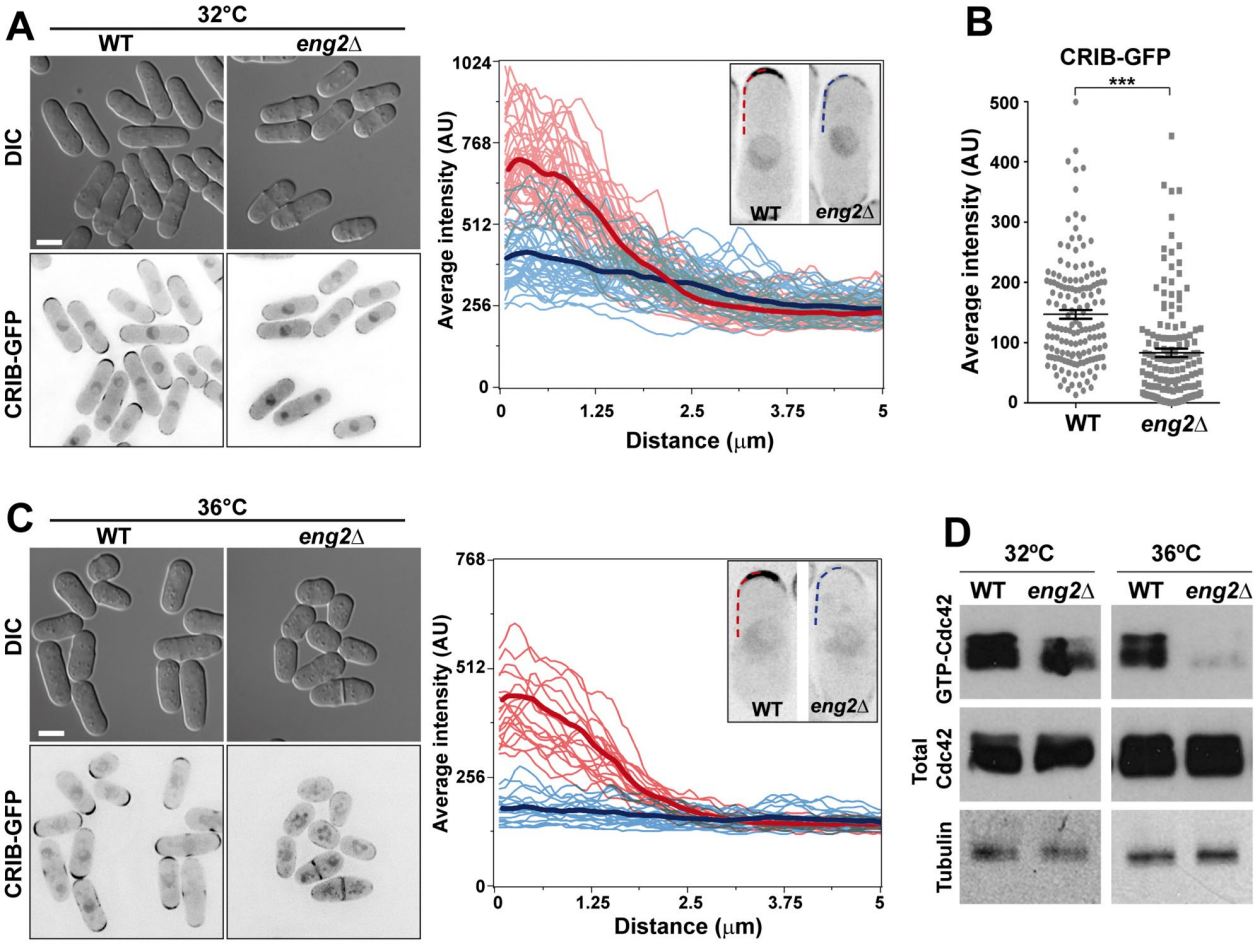
Eng2 regulates the levels of active Cdc42. **(A)** Localization of CRIB-GFP in wild-type and *eng2*Δ cells grown in YES medium at 32 °C. Images show a single focal plane at the center of the cell (DIC) and the maximum intensity projection of 5 focal planes acquired at 0.3-µm intervals. Scale bar, 5 μm. The graph shows plots of the CRIB-GFP intensity along the membrane in wild-type (red) and *eng2*Δ cells (blue). The thick line is the average of > 40 cell tips linescans for each strain. The images show a representative example of the majority of wild-type cells and *eng2*Δ cells. Linescans were done on half tips, aligned at the cell center, as shown by the curved line in the image. **(B)** Average ± s.e.m. intensity of the CRIB-GFP signal at the tips of wild-type and *eng2*Δ cells (n > 150 from 3 independent experiments. ***p-value < 0.0001). **(C)** Localization of CRIB-GFP in wild-type and *eng2*Δ cells grown in YES medium at 36 °C for 10 h. Images show a single focal plane at the center of the cell (DIC) and the maximum projection of 5 focal planes acquired at 0.3-µm intervals. Scale bar, 5 μm. The graph shows plots of the average intensity along the membrane in wild-type (red) and *eng2*Δ cells (blue). The thick line is the average of line scans from 20 cell tips. **(D)** GTP-Cdc42 levels in wild-type and *eng2*Δ mutant cells. HA-cdc42 and HA-cdc42 *eng2*Δ cells were grown at 32 °C and transferred at 32 °C or 36 °C for 3 h. Protein extracts were precipitated with GST-CRIB and blotted with anti-HA antibodies. Total HA-Cdc42 in cell extracts was visualized by western blot. Tubulin was used as loading control.

Polarity establishment in yeasts involves the recruitment and activation of the Cdc42 GTPase to a cortical landmark, where it promotes actin cytoskeleton polymerization and regulates exocytosis reviewed in Refs.^4,12,55,56^. To understand the reasons behind the phenotype of *eng2*Δ cells, we analyzed whether this mutation caused changes in the Cdc42 protein level, localization or activity. Localization of active Cdc42 was visualized in wild-type and *eng2*Δ cells by using the GFP-tagged Cdc42/Rac-interactive binding (CRIB) domain from Gic2 ^57^, which is also used as a marker for Cdc42-GTP in fission yeast ^58^. No significant differences in the monopolar/bipolar distribution of CRIB-GFP were observed between both strains (66% of the wild-type cells versus 61% of *eng2*Δ cells had a bipolar distribution). However, the intensity of the CRIB-GFP signal at the plasma membrane was greatly reduced in the *eng2*Δ mutant as compared to the wild-type strain (Fig. 2A). Measurement of the GFP fluorescence intensity at the cell tips indicated a twofold reduction in *eng2*Δ cells (Fig. 2B). Linescan analysis of the GFP intensity along the cell tips showed that CRIB-GFP failed to concentrate at these areas in *eng2*Δ cells and certain spreading was observed beyond the tip (Fig. 2A). Since *eng2*Δ cells are shorter and rounder when grown at high temperature ^49^, we also analyzed CRIB-GFP distribution at 36 °C. The results showed a higher reduction in the CRIB-GFP signal associated with the tips in the *eng2*Δ mutant (Fig. 2C), suggesting that Eng2 participates in the concentration of active Cdc42 at the tips.

To confirm these observations, the levels of total and active Cdc42 were analyzed in *eng2*Δ cells. Total levels of Cdc42, as detected by immunoblot, were similar in the wild-type and *eng2*Δ strains at both temperatures, ruling out the possibility that this mutant had a defect in Cdc42 stability (Fig. 2D). The amount of active GTP-Cdc42 was determined by pull-down experiments using a Cdc42/Rac interactive binding domain from mammalian Pak2 ^19^ bound to glutathione S-transferase (GST-CRIB). These experiments showed a reduction of GTP-Cdc42 in the *eng2*Δ mutant with respect to wild-type cells, which was more pronounced at high temperature. Together, these results demonstrate that the absence of Eng2 produces a reduction in the cellular pool of active Cdc42 associated with the growing regions.

### Cdc42 oscillation at the tips is reduced in *eng2*Δ mutants

**Figure 3:**
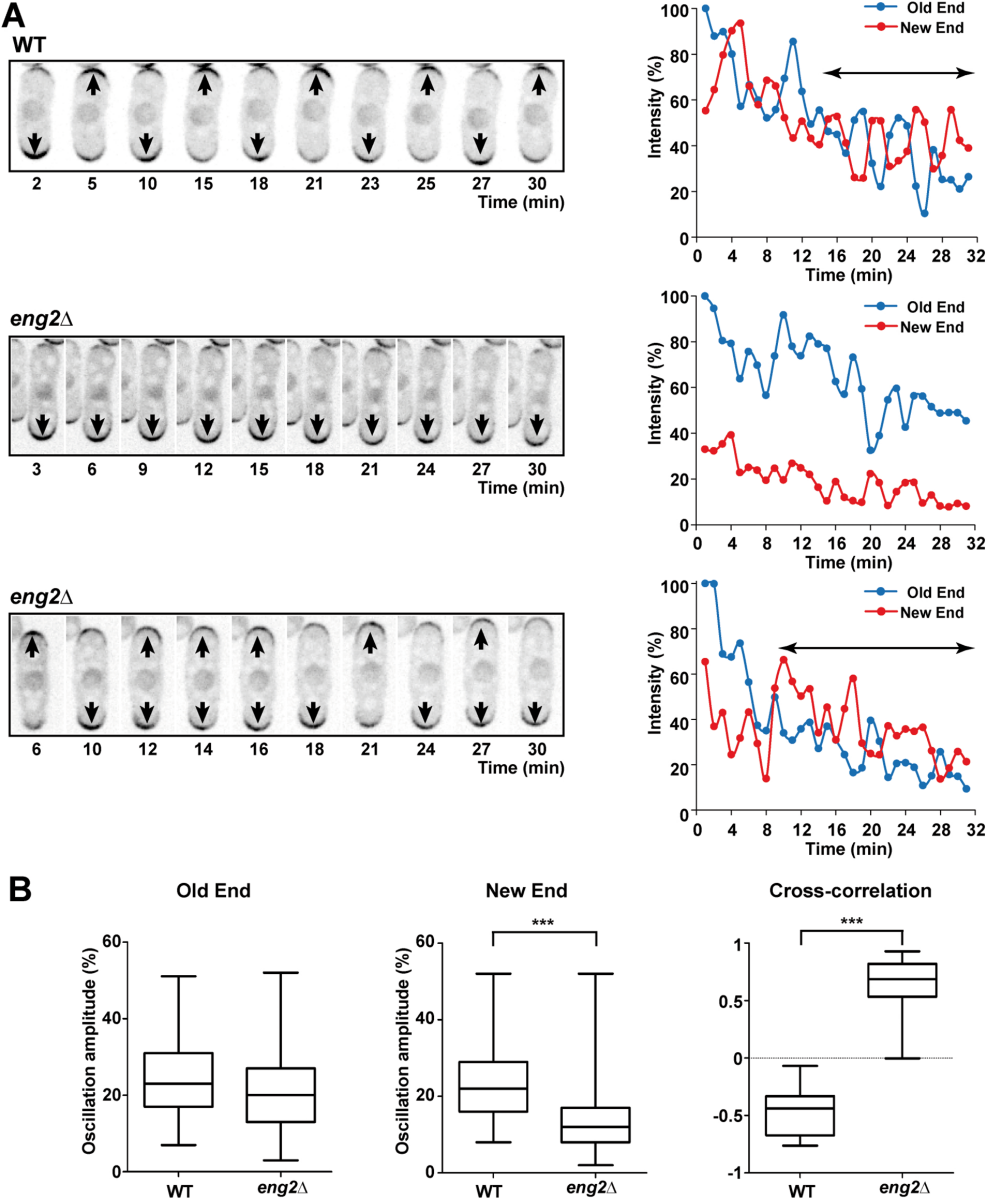
*eng2*Δ mutants show altered oscillations of CRIB-GFP at cells tips. **(A)** Time lapse fluorescence micrographs of wild-type and *eng2*Δ cells expressing CRIB-GFP. Microscopic analysis was performed on IBIDI chambers in YES medium at 32 °C. Images show the maximum intensity projection of 3 focal planes acquired at 0.3-µm intervals, captured at the indicated times (min). Graphs show quantification of the CRIB-GFP fluorescence intensity at the old (blue) and new (red) end in wild type and *eng2*Δ cells. **(B)** Quantification of the oscillation amplitude (n > 100 cells) and cross-correlation (n = 15 cells) of the CRIB-GFP fluorescence signal at the old and new ends in wild type and *eng2*Δ mutant. ***p-value < 0.0001.

Concentration of active Cdc42 exhibit anticorrelated fluctuations and oscillations at polarized cell tips that are essential for NETO ^30^. To analyze whether Cdc42 dynamics were altered in *eng2*Δ mutants, we used time-lapse microscopy in cells carrying CRIB-GFP. The results showed that in wild-type cells Cdc42 had anticorrelated oscillations after the transition from monopolar to bipolar growth, as previously described (Fig. 3A, upper row). In contrast, two types of cells were found in the *eng2*Δ mutant. Monopolar cells had fluctuations of Cdc42 intensity in both tips, although the CRIB-GFP fluorescence intensity at the new pole was always lower than 40% that of the old pole (Fig. 3A, middle row). Bipolar cells had fluctuations and oscillations in both poles, but no clear anticorrelation between them could be observed (Fig. 3A, lower row). Analysis of the oscillation amplitude in old and new ends indicated a significant difference in the new pole between wild-type and *eng2*Δ cells (Fig. 3B). Also, the cross-correlation observed in wild-type cells was completely lost in the *eng2*Δ mutant (Fig. 3B). Therefore, Eng2 is required for the periodic oscillation of active Cdc42 at cell tips and for the anticorrelated fluctuations of both poles that are essential for NETO.

### The N-terminus of Eng2 containing a Pro-rich region, but not the glucanase activity, is required for NETO function

**Figure 4:**
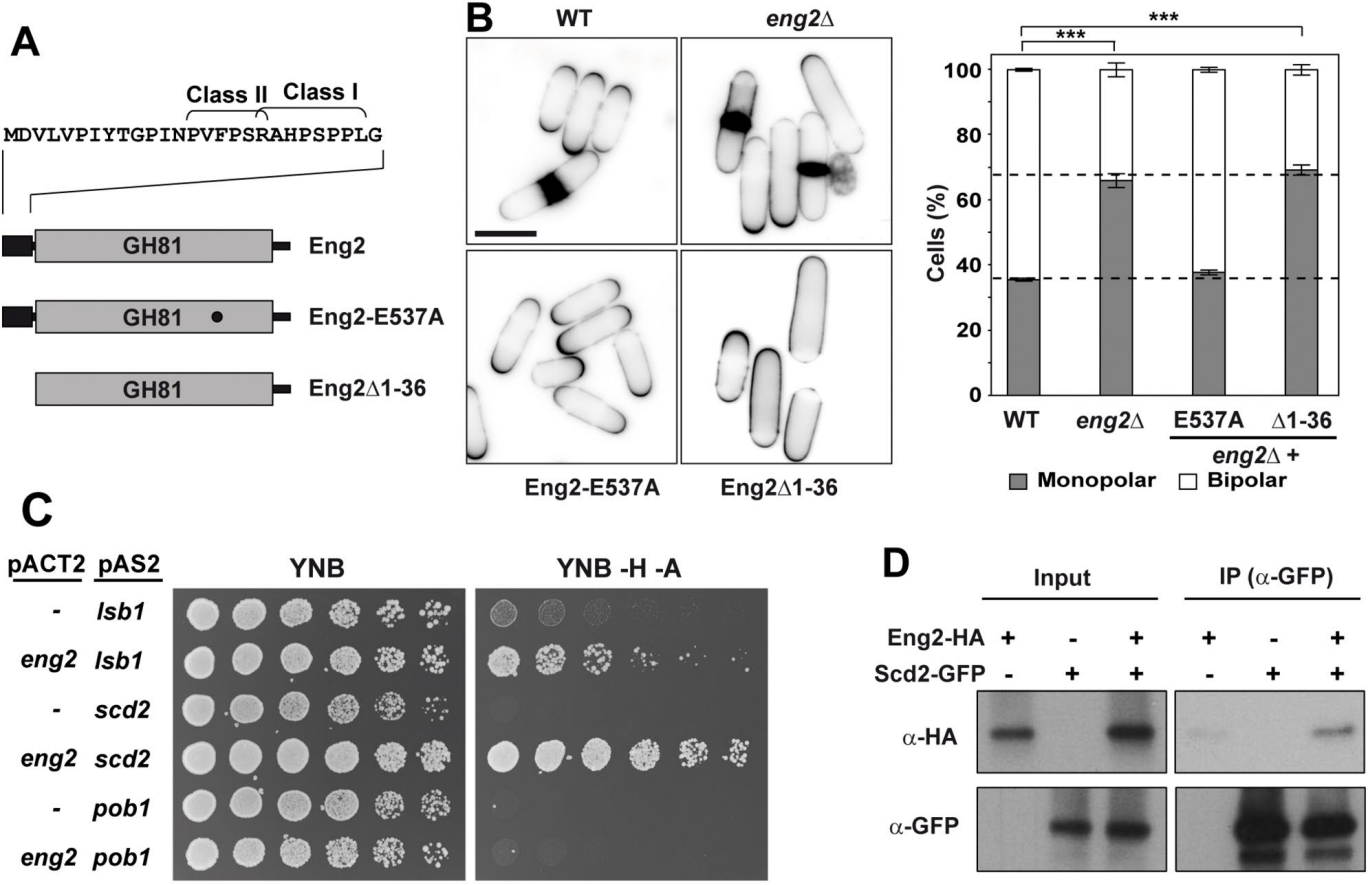
Eng2 interacts with the Cdc42 scaffold protein Scd2 and the glucanase activity is not required for NETO. **(A)** Schematic representation of Eng2 and the *eng2-E537A* and *eng2Δ1-36* mutations. The gray rectangle indicates the Glycoside Hydrolase Family 81 (GH81) domain and the black rectangle marks the Pro-rich region. The putative SH3-interacting Class I (RxxPxxP) and Class II motifs (PxxPxR) are shown. **(B)** Images of calcofluor-stained wild-type and *eng2*Δ cells carrying empty vector (*eng2*Δ) or vectors encoding indicated mutant proteins are shown. Scale bar, 5 μm. The graph to the right shows the average ± s.e.m percentage of monopolar and bipolar cells in wild-type, *eng2*Δ and *eng2*Δ mutant transformed with each construct (n > 150 from 3 independent experiments; ***p-value < 0.0001). **(C)** Two-hybrid analysis of the interaction between Lsb1/Csh3, Scd2 or Pob1 (pAS2) with Eng2 (pACT2). Interaction was assessed by growth on YNB plates without histidine and adenine (YNB -H -A). **(D)** Anti-HA (α-HA) and anti-GFP (α-GFP) immunoblots of total protein extracts (input) or Sepharose-anti-GFP immunoprecipitations (IP (α-GFP)) from cells expressing the endogenous (−) or the indicated Eng2-HA and Scd2-GFP tagged versions (+) under their endogenous promoters.

To determine if the Eng2 glucanase activity was necessary for its function in polarity, we used a strain carrying the *eng2-E537A* allele (Fig. 4A), with a mutation in the catalytic center that destroys the enzymatic activity and produces a protein that is not functional during sporulation ^48,59^. The percentage of bipolar cells of the *eng2*Δ mutant transformed with *eng2-E537A* was similar to that of the wild type strain (Fig. 4B), indicating that Eng2 glucanase activity is not necessary for NETO function.

Eng2 contains a Pro-rich region at the N-terminus in which two consensus motifs (PxxPxR and RxxPxxP) for interaction with SH3 domains are found (Fig. 4A) ^60^. To test whether this region was important for the monopolar/bipolar transition, we generated a truncation allele deleting the first 36 amino acids (*eng2*Δ*1-36*). Interestingly, strains carrying *eng2Δ1-36* had the same phenotype as the *eng2*Δ mutant (Fig. 4B). Immunoblot analysis showed that the abundance of the Eng2-E537A and Eng2Δ1-36 mutant proteins was similar to that of the wild-type protein (Fig. S1C). Therefore, these results were not due to a decrease of Eng2Δ1-36 protein levels and indicate that the first 36 amino acids containing the Pro-rich region are essential for Eng2 function in NETO, whereas the glucanase activity is dispensable.

### Eng2 interacts with the Cdc42 scaffold protein Scd2

Given that the N-terminal Pro-rich region of Eng2 was required for NETO, and that this motif is normally involved in the interaction with SH3 domains, we tested whether Eng2 could interact with some of the Cdc42 regulatory proteins containing SH3 domains by two-hybrid assays. The proteins selected were the Cdc42 scaffold proteins Scd2, which is homologous to Bem1 ^61^, and Pob1, which is homologous to Boi1 and Boi2 from *S*. *cerevisiae* ^19,61,62^. As a positive control we used Lsb1/Csh3, previously found to interact with Eng2 ^49,63^. The results indicated that Eng2 was able to interact with Scd2 but not with Pob1 (Fig. 4C).

To confirm the result of the two-hybrid analysis, co-immunoprecipitation experiments were performed. To this end, Eng2 was tagged with the HA epitope in a strain containing Scd2-GFP. Protein extracts were immunoprecipitated with anti-GFP antibody and the proteins present in the immunoprecipitates were analyzed by immunoblot using anti-GFP or anti-HA antibodies. The results showed that Eng2 was present in the immunoprecipitate (Fig. 4D), confirming the interaction between Scd2 and Eng2.

### Eng2 is required for Scd1 and Scd2 accumulation at the cell tips

**Figure 5:**
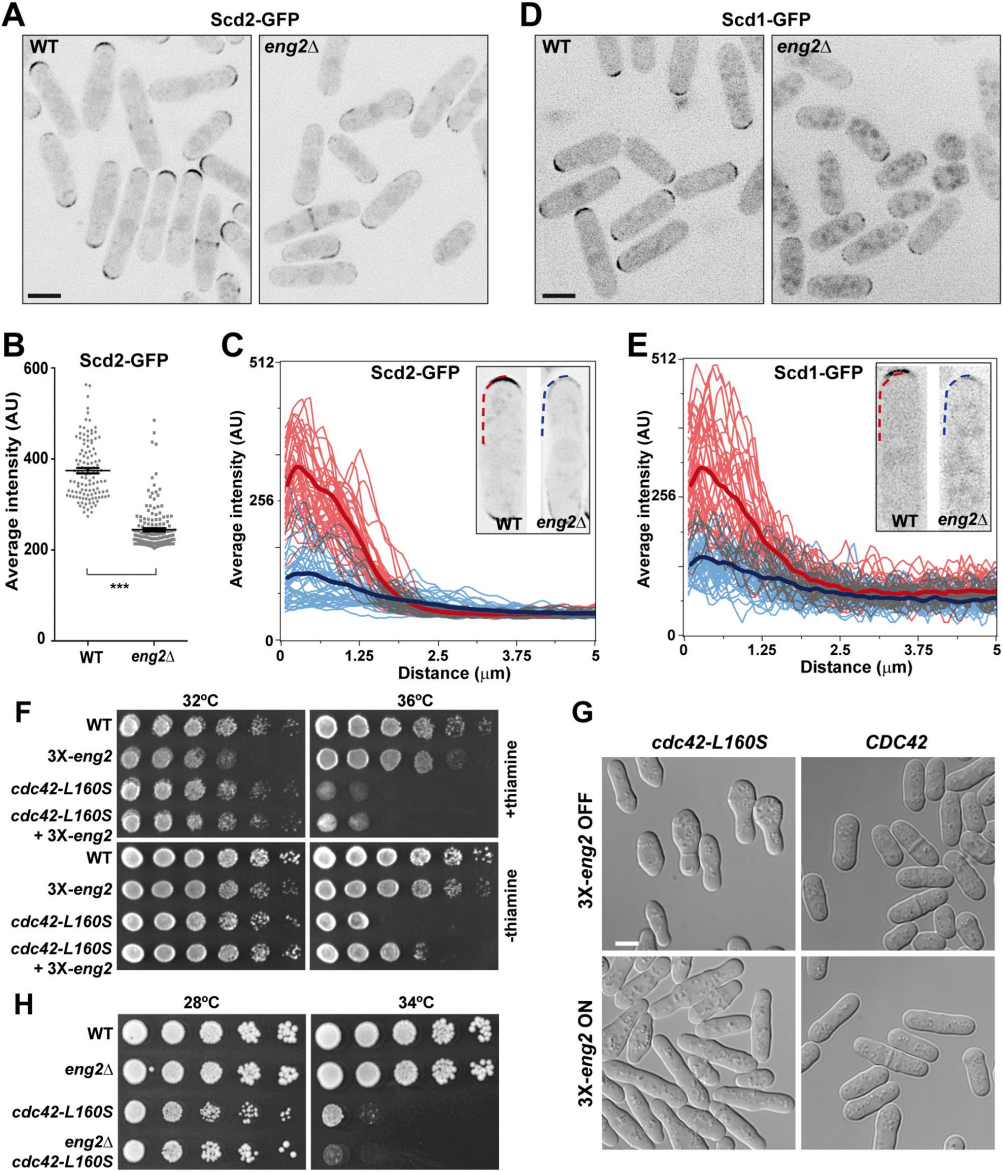
Eng2 governs the intracellular distribution of Scd1 and Scd2. **(A)** Localization of Scd2-GFP in wild-type and *eng2*Δ cells grown in EMM medium at 32 °C. Images show the maximum intensity projection of 5 focal planes acquired at 0.3-µm intervals. Scale bar, 5 μm. **(B)** Average ± s.e.m. intensity of Scd2-GFP at the tips in each strain (n > 150 from 3 independent experiments; ***p-value < 0.0001). **(C)** Plots of the Scd2-GFP fluorescence intensity along the membrane in wild-type (red) and *eng2*Δ cells (blue). The thick line is the average of line scans from > 40 cell tips. The image and graph show a representative example of the majority of wild-type cells and *eng2*Δ cells. Linescans were done on half tips, aligned by the cell center, as shown by the curved line in the image. **(D)** Localization of Scd1-GFP in cells grown in YES medium at 25 °C. Images show the maximum intensity projection of 5 focal planes acquired at 0.3-µm intervals. Scale bar, 5 μm. **(E)** Plots of the Scd1-GFP fluorescence intensity along the membrane in wild-type (red) and *eng2*Δ cells (blue) as in **(C)**. **(F)** Serially diluted growth spots of the wild-type (WT) and the *cdc42-L160S* either carrying *Pnmt1-eng2*^+^ (3X-eng2) thiamine repressible version or not, grown in EMM medium in the presence (+ thiamine) or absence (-thiamine) of thiamine at the indicated temperatures. **(G)** DIC micrographs showing the morphology of *CDC42* and *cdc42-L160S* cells overexpressing *eng2*^+^ (3X-eng2 ON) from the Pnmt1 promoter or not (3X-eng2 OFF). Scale bar, 5 μm. **(H)** Serially diluted growth spots of the wild-type, *eng2*Δ, *cdc42-L160S* and *eng2*Δ *cdc42-L160S* mutants at the indicated temperatures in YES medium.

The above results prompted us to analyze Scd2 levels and localization in wild-type and *eng2*Δ cells. As previously described ^64^, Scd2 localized to the growing tip in both strains (Fig. 5A). However, the intensity of Scd2-GFP signal at the growing tips in *eng2*Δ cells was greatly reduced in comparison to control cells (Fig. 5B), although the amount of total Scd2 protein was not changed (Fig. S1D). Linescan analysis of the fluorescence intensity along the membrane showed that Scd2 was less concentrated at the tips in *eng2*Δ cells than in wild-type cells (Fig. 5C). In addition, we did observed certain spreading beyond the tip of the weak Scd2 signal in *eng2Δ* cells. Scd2 is a scaffold protein which facilitates Cdc42 activation by the Scd1 GEF ^27,61,62^. The localization of Scd1 and Scd2 is interdependent and it is also mediated by Gef1 ^27,28^. Therefore, localization of Scd1-GFP was also analyzed in both strains. The intensity of Scd1-GFP at the cell tips was also reduced in *eng2*Δ cells as compared to control cells (Fig. 5D,E). Thus, these results indicate that Eng2 is involved in the regulation of the intracellular distribution of Scd1 and Scd2, likely by binding to Scd2.

Overexpression of Scd1 is able to restore the ability to grow at 36 °C of the thermosensitive *cdc42-879* strain carrying the *cdc42-L160S* allele ^19,23^. Since Eng2 participates in the membrane localization of Scd1 and in the activation of Cdc42 at the growing tips, we tested whether *eng2*^+^ over-expression could also suppress the thermosensitive growth of this strain. Indeed, overexpression of *eng2*^+^ using the strong inducible P_*nmt1*_ promoter partially complemented the growth defect of the strain carrying the *cdc42-L160S* allele at high temperature (Fig. 5F). Moreover, *cdc42-L160S* expressing cells had a variety of cell shapes, including pear-like cells, lemon-shaped cells, and round cells ^19^, and these morphological defects were largely corrected by over-expression of *eng2*^+^ (Fig. 5G). Reciprocally, deletion of *eng2*^+^ slightly exacerbated the thermosensitive phenotype of *cdc42-L160S* cells (Fig. 5H).

## Discussion

Polarized growth controls important events like morphogenesis, cell motility, embryogenesis, and stem cell differentiation ^4,5,21,24,65^. The master regulator of cell polarization is Cdc42, a highly conserved member of the Rho family GTPases. In this work, we demonstrate that the “moonlighting protein” Eng2 that acts as a glucanase during sporulation and as a component of the endocytic machinery in vegetative cells ^48,49^, also plays a role in polarized growth, regulating Cdc42 activation and dynamics. Our results suggest that Eng2 is necessary for growth activation at the new end (NETO) in fission yeast. This is based on robust data showing that the *eng2*Δ mutant has defects in the transition from monopolar to bipolar growth, with most of the cells growing at the old end and never activating growth at the new end. The main polarity markers like Tea1 and Pom1 did not have localization defects in the *eng2*Δ background, and the monopolar growth pattern of *tea1*Δ mutants, with one cell growing at the old end and the other at the new end was different from that of *eng2*Δ cells. Our results suggest that Eng2 is not a polarity marker required to establish polarized growth but can be described as a new member of the Cdc42 pathway that maintains polarized growth acting downstream of Tea1 and Tea4 ^14,66,67^.

Curiously, *bud6*Δ mutant presents a monopolar growth pattern similar to *eng2*Δ cells, with most of the cells growing only at the old end ^51^. Bud6 is a protein that collaborates with Cdc42 in the activation of For3 by relieving its autoinhibition ^18^. Consistent with the view that Eng2 role is to regulate Cdc42 activation, we found that Eng2 facilitates Scd1–Scd2 localization, Scd1 being a GDP–GTP exchange factor for Cdc42 ^25^, and Scd2 a multi-domain adaptor protein that acts as a scaffold to support Scd1–Cdc42 interaction and regulates cell polarity in fission yeast^28,61^. Two-hybrid analysis and co-immunoprecipitation experiments unveiled that Eng2 interacts with Scd2. This protein contains two SH3 domains near the N-terminus, which usually mediate assembly of specific protein complexes by binding to proline-rich peptides ^68^. It is likely that the Eng2–Scd2 interaction is mediated by the SH3 domains of Scd2 and the proline-rich region of Eng2, since deletion of this region caused polarized growth defects similar to those caused by depletion of *eng2*^+^. Previously, we demonstrated that the proline-rich region of Eng2 interacts with Lsb1/Csh3, which also contains SH3 domains, and that this interaction is essential for Eng2 function in endocytosis ^49^. To our knowledge, Eng2 is the first endocytic protein identified in *S. pombe* that physically interacts with a component in the functional vicinity of Cdc42, although previous reports have suggested a possible relation between endocytic proteins and Cdc42 effectors. This is the case of the Pak1 kinase, which is part of a Cdc42 ternary complex that phosphorylates the endocytic Myosin Myo1 or the F-BAR protein Cdc15, which contributes to Gef1 recruitment to the new end for NETO^69,70^. Interestingly, endocytic machinery components capable of interacting with polarity factors have been previously identified in *S. cerevisiae*. This is the case of the ENTH domain of the yeast epsins, which is essential for viability in *ent1*Δ *ent2*Δ cells and binds to Cdc42 GAP proteins like Rga1, Bem3 or Rga2. ENTH1^Y100R^ mutation that disturbs ENTH’s role in the cell can be bypassed by Gic1, Gic2 or Bem1 overexpression, but not by others effectors like Ste20, Cla4 or Bni1. Additionally, while this manuscript was under revision, it was reported that the Ecm25 protein associates with polarized endocytic sites and interacts with the polarity regulator Cdc42 and several late-stage endocytic proteins ^71^. Now, we show that *eng2*^+^ overexpression recover morphological and growth defects in the *cdc42-L160S* allele through Cdc42 re-localization to the ends of the cell. Additionally, we demonstrate that Eng2 binds to the multi-domain adaptor protein Scd2. Thus, we propose that Eng2 is necessary for Scd1–Scd2 localization and it facilitates Cdc42 activation.

A key observation derived from our work is that Cdc42 activation and dynamics are dependent on Eng2. Depletion of *eng2*^+^ caused not only CRIB-GFP delocalization but also a reduction in the total active Cdc42 in the cells, especially at high temperature. Supporting the idea that Eng2 is a new component involved in Cdc42 regulation, we also detected genetic interactions with the *cdc42-L160S* allele. Finally, we provide evidences suggesting that Eng2 controls oscillations in Cdc42 activation associated to bipolar growth. In the absence of Eng2, the oscillation amplitude decreased in the new end and the cross-correlation between the two ends was lost. These defects probably reflect alterations in the positive and negative feedback loops regulating the polarity of Cdc42 activation ^72^. Cellular oscillations control important aspects of cell physiology, such as polarized growth, DNA synthesis, mitosis and development. In yeast, these oscillations have been identified as a method to break the cellular symmetry ^73^ or to maintain bipolar growth ^30^, but these oscillations are also presents in plants, insects or animal cells, ensuring pollen tube growth ^74^, tissue morphogenesis ^75^ or supporting the development of somites in vertebrate embryos ^76,77^.

In conclusion, our work unveils a previously unnoticed contribution of the β(1,3)-glucanase Eng2 to the establishment of polarized growth. Altogether, we describe a component of the endocytic machinery that also impinges on the Cdc42-dependent polarized growth pathway in fission yeast, being necessary for Scd1–Scd2 tip localization and for Cdc42 activation and dynamics to sustain bipolar growth.

## Materials and methods

### Yeast strains and growth conditions

See Supplementary Table S1 for strain information. Yeast cells were grown on YES medium or minimal medium (EMM) with the required supplements ^78^. Construction of yeast strains was performed using standard procedures: either integration of cassettes or genetic crossing. For overexpression experiments using the *nmt1*^+^ promoter, cells were grown in EMM containing 15 μM thiamine up to the logarithmic phase. Then, the cells were harvested, washed three times with EMM, and inoculated in fresh medium (without thiamine) at an OD_595_ = 0.05. Synchronization of strains carrying the thermosensitive *cdc10-129* mutation was achieved by growing the cells at the permissive temperature (25 °C) to early log phase (OD_595_ = 0.5) and then shifting the cultures to 36 °C for 4 h. Cells were released from arrest by transfer to 25 °C, and samples were taken at different times after release.

### Plasmid constructions

Plasmid pJED21 contained *eng2Δ1-36-mCherry* was constructed by cloning a *BamHI-SpeI* fragment obtained from pJED14 that contained the C-terminal region of *eng2*^+^ and mCherry into the corresponding sites of pJED20 (Details about pJED20 plasmid construction can be consulted in Ref.^49^). Plasmid pJED22 contained the *eng2-E537A-mCherry* allele was constructed by recombinant PCR, generating the desired substitution in a *Bam*HI-*Spe*I fragment, which was cloned into plasmid pJED14 (Details about pJED14 plasmid construction can be consulted in Ref.^49^).

For two-hybrid analysis, *pob1*^+^ ORF was cloned into the *NdeI-SmaI* sites of pAS2. *scd2*^+^ ORF was cloned into the *BamHI* sites of pAS2. The *lsb1*/*csh3*^+^ ORF was cloned into the *Sma*I sites of pAS2. *eng2*^+^ ORF was cloned into the *Sma*I sites of pACT2. *Saccharomyces cerevisiae* AH109 (*MATa, trp1-901, leu2-3, 112, ura3-52, his3-200, gal4Δ, gal80Δ, LYS2::GAL1*_*UAS*_*-GAL1*_*TATA*_*-HIS3, GAL2*_*UAS*_*-GAL2*_*TATA*_*-ADE2, URA3::MEL1*_*UAS*_*-MEL1*_*TATA*_*-lacZ*) was transformed and grown on plates without leucine, tryptophan, and histidine and supplemented with 40 mM 3-aminotriazole.

### Western blotting

For immunoblotting, cells were grown to mid-log phase and cells were collected and resuspended in 5 ml of buffer E (50 mM sodium citrate, 100 mM sodium phosphate, pH 6.0, and 0.8 M sorbitol). Protoplasts were generated by incubation with Glucanex (1 mg/ml; Sigma-Aldrich) and Zymolyase-100 T (3 mg/ml; ICN Biomedicals) for 1 h. Then, the protoplasts were resuspended in lysis buffer (50 mM Tris, pH 8, 150 mM NaCl, 1% Triton X-100, 1 mM DTT, 1 mM PMSF, 1 μg/ml aprotinin, 1 μg/ml leupeptin, 1 μg/ml pepstatin). Protein concentrations were determined using the BioRad Protein Assay kit (BioRad). 50 μg of protein extracts were resolved by SDS–polyacrylamide gel electrophoresis (PAGE) on 10% gels and probed with anti-GFP (JL-8 Living Colors, Clontech), polyclonal DsRed (Living Colors, Clontech), anti-HA Rat monoclonal antibody (3F10, Roche) or anti-tubulin (Sigma 75168). Protein transfer, blotting, and chemiluminescence detection were performed using standard procedures ^49^.

### Immunoprecipitation Eng2-Scd2

For immunoprecipitation of Scd2-Eng2, cells were grown to mid-log phase then were crosslinked with 1% formaldehyde for 10 min at 30 °C. The reaction was quenched by adding glycine to 250 mM and incubating for 5 min on ice. Cells were collected, washed and broken with glass beads in lysis buffer (25 mM Tris HCl, pH 7.5, 150 mM NaCl, 0.5% SDS, 1% NP40, 1 mM PMSF, 1 μg/ml aprotinin, 1 μg/ml leupeptin, 1 μg/ml pepstatin). Clarified extracts were immunoprecipitated by adding 10 μl of GFP-Trap beads (Chromotek) for 1 h at 4 °C.The beads were washed six times with lysis buffer and resuspended in sample buffer. Samples were resolved by SDS-PAGE on 10% gels and probed with anti-GFP (JL-8 Living Colors, Clontech) and anti-HA (Anti-HA-Peroxidase High Affinity 3F10, Roche). Protein transfer, blotting, and chemiluminescence detection were performed using standard procedures.

### Glutathione–sepharose (GS) pull-down and immunoprecipitation

The expression vector pGEX-Cdc42/Rac interactive binding (CRIB) ^79^ was used to transform *E. coli* and produce GST fused to the mammalian Pak2 binding domain for Cdc42. The fusion protein was produced according to the manufacturer’s instructions and immobilized on GS beads. The amount of GTP-bound Cdc42 was determined using a pull-down assay as described previously ^26^. In brief, extracts from Wild-Type or *eng2*Δ strains carrying integrated HA-cdc42 were obtained by using 500 μl of lysis buffer (50 mM Tris, pH 7.5, 20 mM NaCl, 0.5% NP-40, 10% glycerol, 0.1 mM dithiothreitol, 1 mM NaF and 2 mM Cl_2_Mg, containing 100 μM PMSF, leupeptin, and aprotinin). Cell extracts (2 mg of total protein) were incubated with 10 μg of GST–CRIB protein coupled to GS beads for 2 h, washed four times, and blotted with anti-HA antibody. Total HA-Cdc42 levels in whole-cell extracts (50 μg of total protein) were monitored by Western blot. Tubulin was used as loading control.

### Microscopy

Samples were observed in wet preparations with a Personal Deltavision (Applied Precision, LLC) microscope equipped with a CoolSNAP HQ2 (Photometrics) camera and controlled with softWoRx Resolve 3D. Depending on the experiment, a single focal plane at the centre of the cell or a stack of 8–16 images covering the entire volume of the cell (z-series) with a spacing of 0.3–0.6 μm were captured. Stacks were deconvolved with softWoRx Resolve 3D and the maximum projection was generated.

Calcofluor white (Blankophor BBH, Bayer Corporation) staining was performed by adding 2 μl of a stock solution (10 mg/ml) to 100 μl of samples for 1 min, followed by a wash with phosphate buffer saline (PBS). Actin staining was performed with Alexa Fluor 488 phalloidin as previously described ^80^. Cells were fixed with 4% formaldehyde (EM-grade MeOH-Free, Polysciences) in PM buffer (35 mM K_2_HPO_4_, pH 6.8, 0.5 mM MgSO_4_) for 1 h at 25 °C. Then, cells were washed with PM buffer and permeabilized with PM buffer containing 1% Triton X-100 for 30 s. After three washes with PM buffer, 5 μl of Alexa Fluor 488 phalloidin (Molecular Probes) was added to 2 μl of cells and these were incubated for 1 h in the dark.

To analyze active Cdc42 oscillations at the tips, time-lapse experiments were performed with wild-type and *eng2*Δ cells expressing CRIB-GFP. Cells were placed in μ-Slide eight-wells coated with soybean lectin and imaged every 1 min over a 45-min period using a spinning-disk confocal microscope. Bipolar cells were selected (according to cell length), and the average CRIB-GFP intensity at the tips was measured using ImageJ software. Intensities from both tips were normalized to the maximum intensity and plotted against the time. Period and amplitude between oscillations were calculated from the plots, the period being the time between two subsequent maxima of intensity, and the amplitude being the difference between maximum and minimum intensities within the oscillation ^81^.

The cross-correlation coefficient was calculated to compare the correlation between two different matrixes, the fluorescence intensity in the old end “*versus*” the fluorescence intensity in the new end, both in WT and *eng2*Δ mutant cells. The Pearson correlation coefficient (*p*) was determined according to the formula:

$$\rho_{x,y} = \frac{Cov(X,Y)}{{\sigma_{x} ,\sigma_{y} }},$$

where, *Cov* is the covariance, *σx* is the standard deviation of *X* and *σY* is the standard deviation of *Y*.

## Supplementary Information

Supplementary Information.
