# Supplementary material for: Eng2, a new player involved in feedback loop regulation of Cdc42 activity in fission yeast
Source: Sci Rep. 2021 Sep 9;11:17872. doi: 10.1038/s41598-021-97311-6 (PMC8429772; doi:10.1038/s41598-021-97311-6)
Supplement: Supplementary file 1 — Supplementary Information. [file 41598_2021_97311_MOESM1_ESM.pdf]

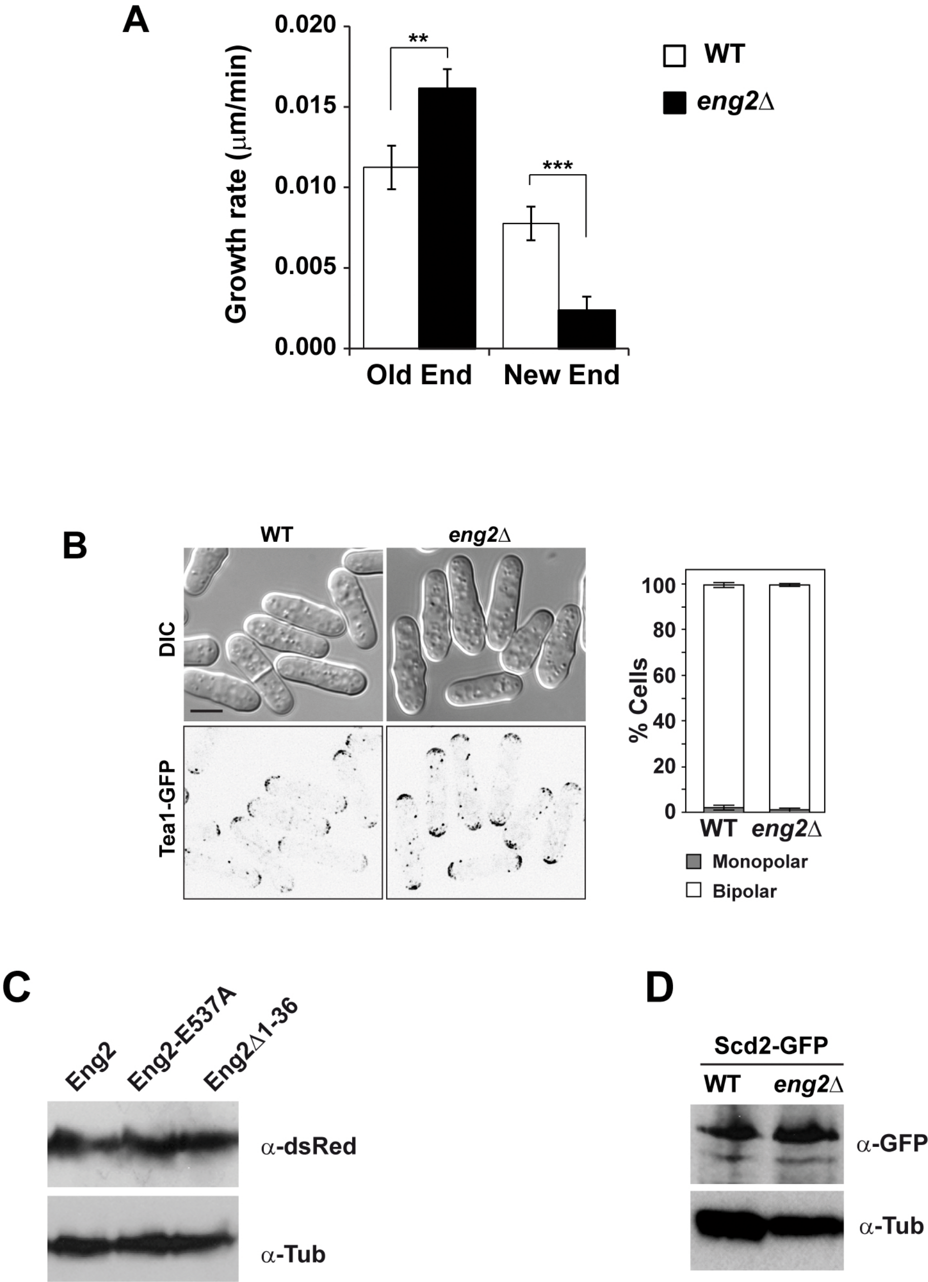
A
Growth rate (μm/min)
0.020
0.015
0.010
0.005
0.000
**
***
Old End
New End
WT
eng2Δ
B
WT
eng2Δ
DIC
Tea1-GFP
% Cells
100
80
60
40
20
0
WT
eng2Δ
Monopolar
Bipolar
C
Eng2
Eng2-E537A
Eng2Δ1-36
α-dsRed
α-Tub
D
Scd2-GFP
WT
eng2Δ
α-GFP
α-Tub

SUPPLEMENTARY FIGURE LEGENDS

Figure S1. Eng2 is required for proper localization of proteins required for polarized growth. (A) Growth rate at the old end and new end of wild-type (n = 14 cells) and *eng2Δ* (n = 32 cells) strains**. (B)** Localization of Tea1-GFP in wild-type and *eng2*Δ cells. Fluorescence and DIC images are shown. The graph represents the average ± s.e.m. percentage of monopolar or bipolar cells in each strain of 3 independent experiments (n>150). Scale bar, 5 µm. **(C).** Anti-dsRed and anti-Tubulin immunoblot analysis of total protein extracts from *eng2*Δ cells expressing either the wild type Eng2 or the Eng2-E537A and Eng2Δ1-36 mutant proteins. Tubulin was used as loading control. **(D)** Anti-GFP immunoblot of total protein extracts from wild-type and *eng2*Δ cells expressing Scd2-GFP. Tubulin was used as loading control.

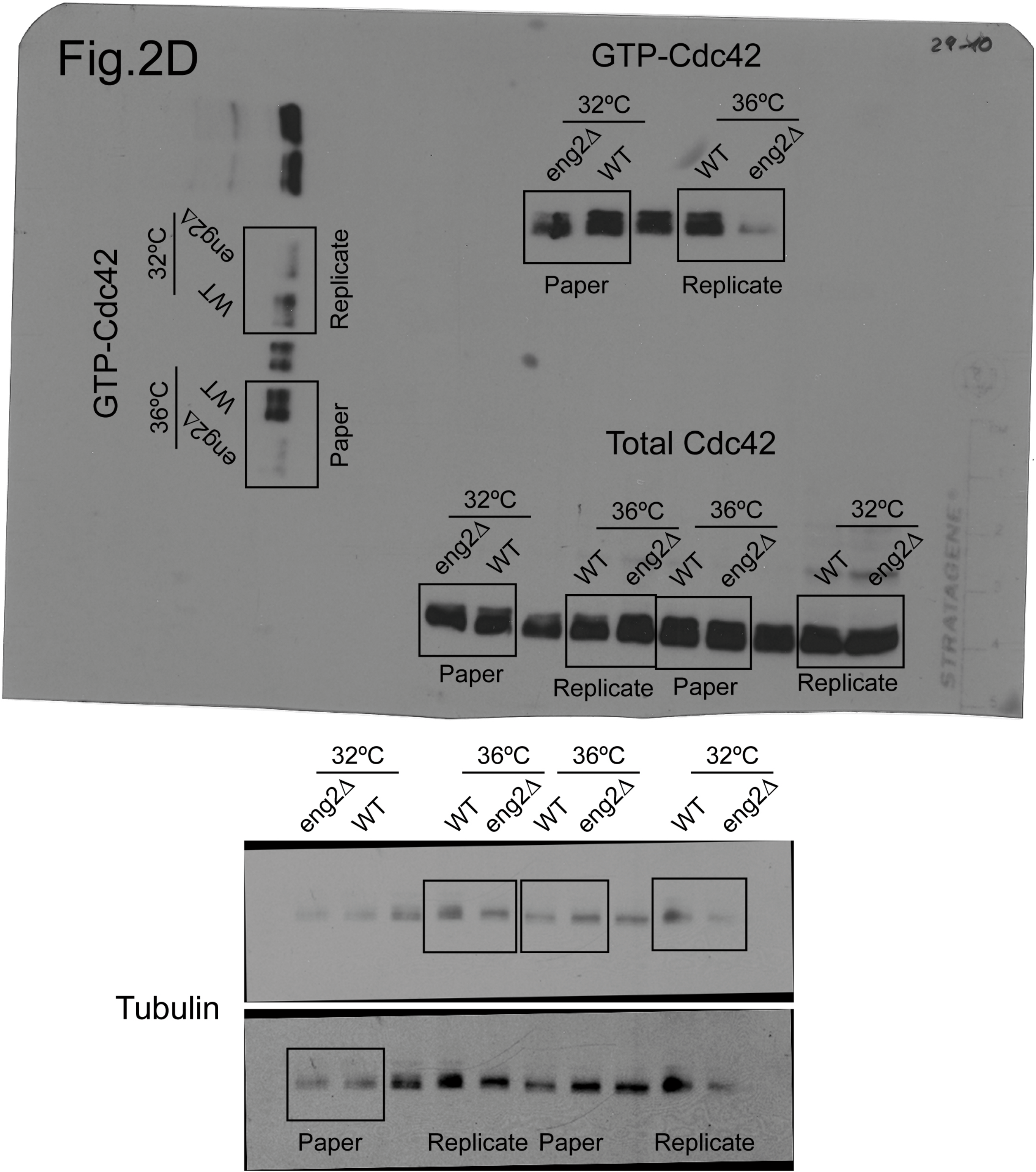

Fig.2D
GTP-Cdc42
32ºC
36ºC
eng2Δ
WT
WT
eng2Δ
Paper
Replicate
GTP-Cdc42
32ºC
eng2Δ
WT
Replicate
36ºC
WT
eng2Δ
Paper
Total Cdc42
32ºC
eng2Δ
WT
36ºC
WT
eng2Δ
36ºC
WT
eng2Δ
32ºC
WT
eng2Δ
Paper
Replicate
Paper
Replicate
32ºC
eng2Δ
WT
36ºC
WT
eng2Δ
36ºC
WT
eng2Δ
32ºC
WT
eng2Δ
Tubulin
Paper
Replicate
Paper
Replicate

Fig.4D

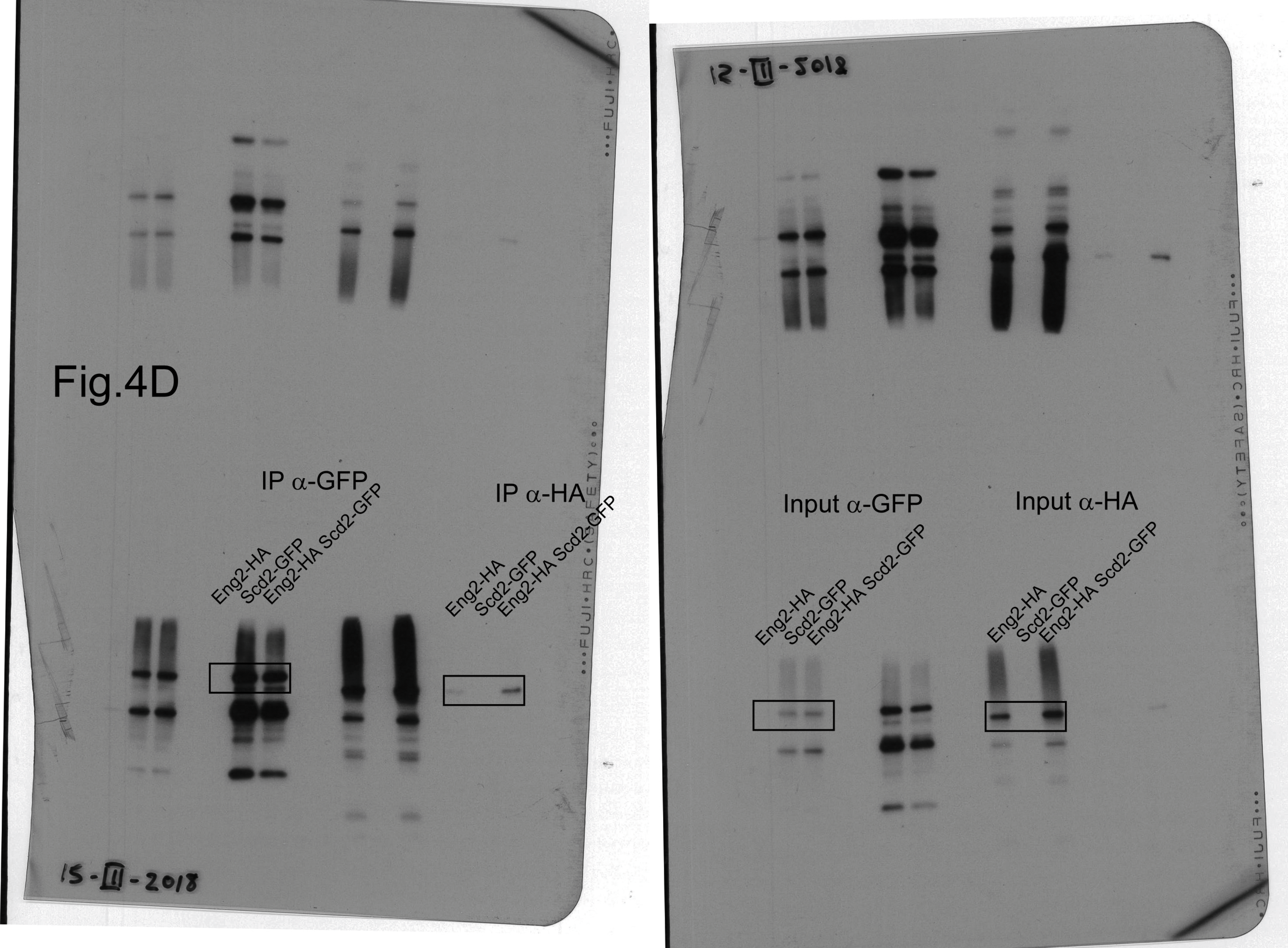
IP α-GFP
Eng2-HA
Scd2-GFP
Eng2-HA Scd2-GFP
IP α-HA
Eng2-HA
Scd2-GFP
Eng2-HA Scd2-GFP
15-III-2018
12-III-2018
Input α-GFP
Eng2-HA
Scd2-GFP
Eng2-HA Scd2-GFP
Input α-HA
Eng2-HA
Scd2-GFP
Eng2-HA Scd2-GFP

Fig.S1C

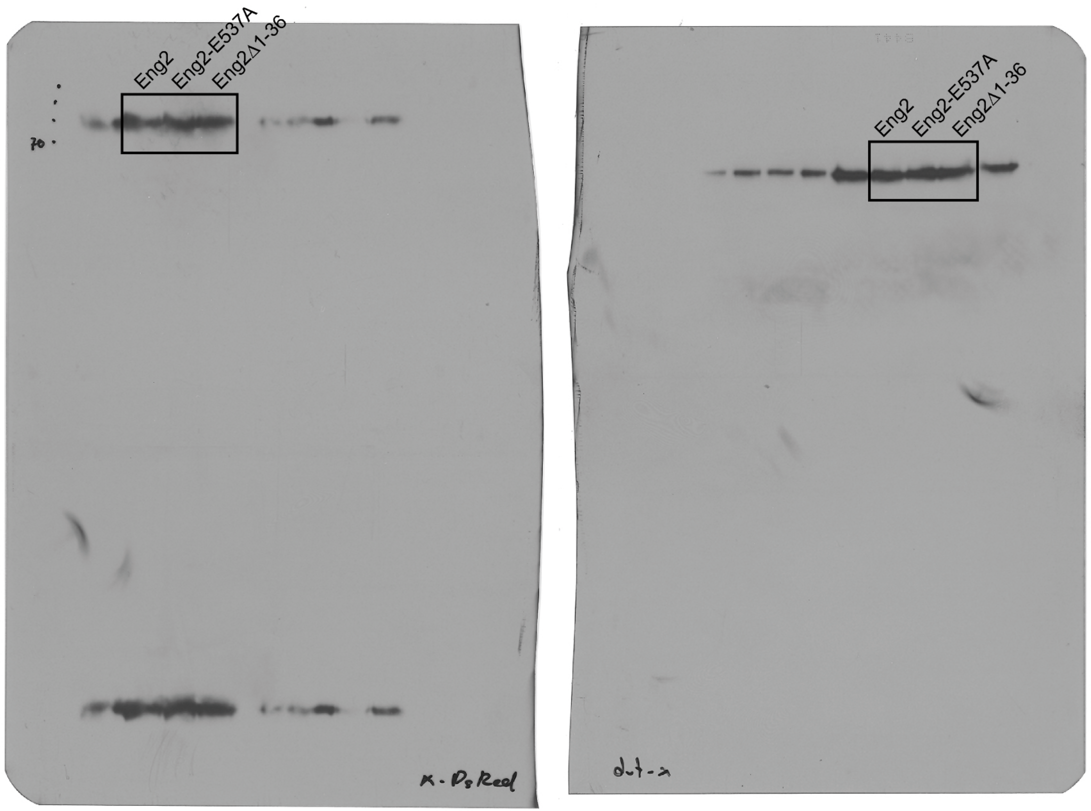

Fig.S1D

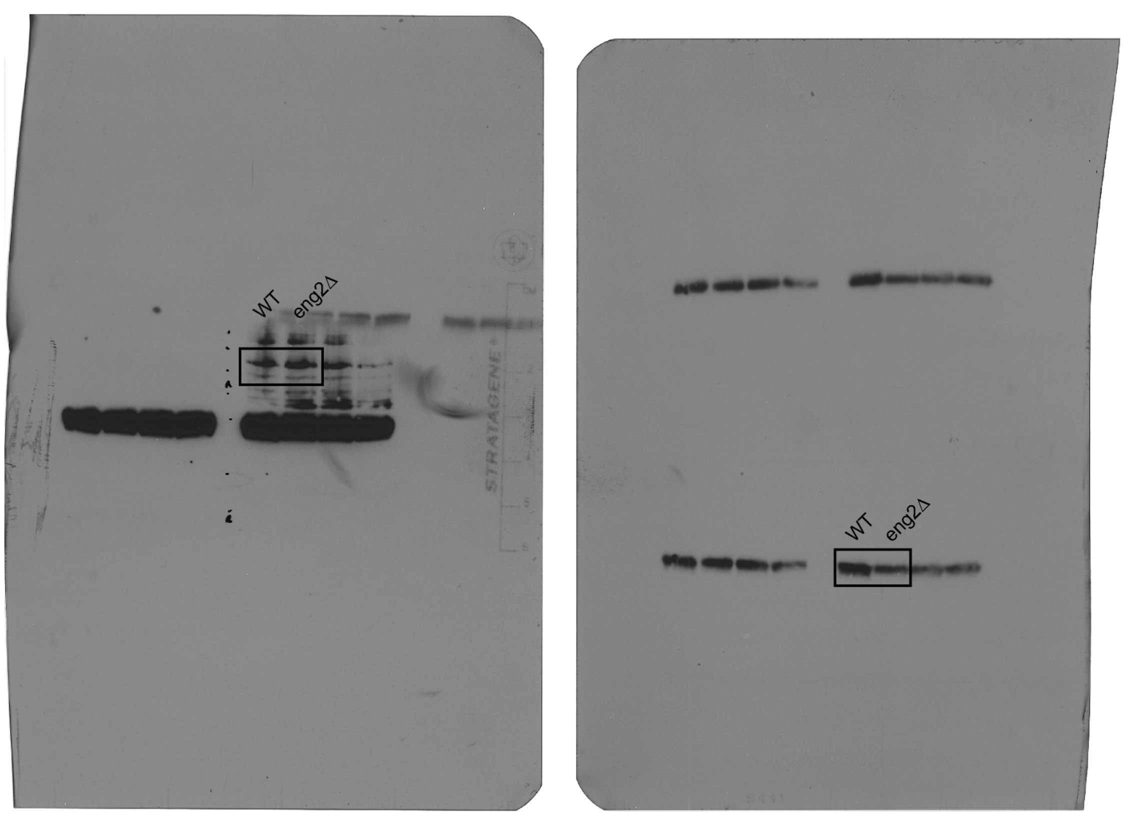

Supplementary Table 1. Strains used.

**Strain**	**Genotype**	**Origin**
h123	*ura4-Δ18 h*$^{-}$	Lab stock
OL821	*cdc10-129 leu1-32 h*$^{-}$	Lab stock
OL891	*myo52-GFP::kanMX4 leu1-32 ura4-Δ18 his3-Δ1 h*$^{-}$	Lab stock
OL869	*tea1-GFP::kanMX4 leu1-32 ura4-Δ18 h*$^{+}$	Lab stock
OL1807	*CRIB-GFP::ura4+ leu- h+*	Lab stock
OL2630	*scd1-GFP::kanMX4 leu1-32 ura4-Δ18 h+*	Lab stock
PPG5668	*scd2-GFP::kanMX4 leu1-32 ura4-Δ18 h+*	Lab stock
PPG4711	*HA-cdc42:ura4+ leu1-32 ura4-Δ18 ade6-M216 h+*	Rincón SA, 2009
PPG6521	*HA-cdc42-L160S::ura4+ leu1-32 ura4-Δ18 h-*	Rincón SA, 2009
561	*bgs4Δ::ura4*$^{+}$ P*bgs4*$^{+}$*::GFP-bgs4*$^{+}$*:leu1*$^{+}$ *leu1-32 ura4-Δ18 his3-Δ1 h*$^{-}$	Cortés JC, 2005
OL24	*ura4-Δ18 eng2Δ::ura4*$^{+}$ *h*$^{-}$	Encinar del Dedo J, 2009
OL759	*ade6 M210 leu1-32 ura4.Δ18 eng2::KanMX4 h-*	Encinar del Dedo J, 2009
OL1198	*ade6 M210 leu1-32 ura4.d18 eng2-HA:KanR h-*	Encinar del Dedo J, 2014
OL831	*cdc10-129 leu1-32 eng2Δ::kanMX4 h*$^{-}$	This study
OL909	*myo52-GFP::kanMX4 eng2Δ::ura4*$^{+}$ *leu1-32 ura4-Δ18 his3-Δ1 h*$^{-}$	This study
OL871	*bgs4Δ::ura4*$^{+}$ P*bgs4*$^{+}$*::GFP-bgs4*$^{+}$*:leu1*$^{+}$ *eng2Δ::kanMX4 leu1-32 ura4-Δ18 his3-Δ1 h*$^{+}$	This study
OL873	*tea1-GFP::kanMX4 eng2Δ::ura4*$^{+}$ *leu1-32 ura4-Δ18 h*$^{+}$	This study
OL1808	*CRIB-GFP::ura4+ eng2Δ::kanMX4 h?*	This study
OL2610	*HA-cdc42::ura4+ eng2Δ::kanMX4 h?*	This study
OL2619	*scd2-GFP::kanMX4 eng2Δ::ura4+ h?*	This study
OL2621	*scd2-GFP::kanMX4 eng2-HA::KanMX4 h?*	This study
OL2631	*scd1-GFP::kanMX4 eng2Δ::ura4+ leu1-32 ura4-Δ18 h?*	This study
OL984	*Pnmt1_3x_eng2:kanMX4 ura4-Δ18 h-*	This study
OL1166	*HA-cdc42-L160S::ura4+ eng2Δ::kanMX4 h?*	This study
OL1668	*HA-cdc42-L160S::ura4+ Pnmt1_3x_eng2:kanMX4 h?*	This study
